# Outcomes and Safety of Catheter Ablation in the Elderly

**DOI:** 10.19102/icrm.2025.16084

**Published:** 2025-08-15

**Authors:** Khalid Sawalha, Anis John Kadado, Shayal Pundlik, Kyle Gobeil, Mohamed Abdelazeem, Fadi Chalhoub

**Affiliations:** 1 Department of Cardiology, UMass Chan Medical School – Baystate, Springfield, MA, USA; 2 Department of Cardiology, Yale University, New Haven, CT, USA

**Keywords:** Catheter ablation, elderly, octogenarians, outcomes

## Abstract

Catheter ablation has emerged as a first-line therapy for many arrhythmias. However, data on the safety and outcomes of catheter ablation in the elderly population remain limited. Here, we aimed to study the outcomes of catheter ablation in octogenarians. The data used in this study were obtained from the National Inpatient Sample database through years 2016–2019. We identified patients ≥80 years old who were diagnosed with atrial fibrillation (AF), atrial flutter (AFL), supraventricular tachycardia (SVT), or ventricular tachycardia (VT) as primary diagnoses. The patients’ characteristics and common procedure complications were extracted. We investigated the predictors of mortality and in-hospital complications using multivariable logistic regression. A total of 18,595 patients were included in our analysis. The most common procedure performed was ablation for AF (46%), followed by AFL ablation (23%), VT ablation (18%), and SVT ablation (12%). Higher rates of tamponade (1.6%) were seen in patients undergoing VT ablation. A Charlson’s comorbidity index (CCI) score of ≥3 points was used as an independent predictor for complications (odds ratio [OR], 2.14; 95% confidence interval [CI], 1.4–3.3, *P* = .001). Mortality was higher in VT ablation (4.2%) compared to AFL (1.3%), AF (0.9%), and SVT (0.3%). After logistic regression analysis, a CCI score of ≥3 points (OR, 14.7; 95% CI, 1.88–114.9; *P* = .01) and tamponade (OR, 4.9; 95% CI, 1.65–14.8; *P* = .004) were independent predictors of mortality. We found a low incidence of procedural complication rates across all ablation groups in octogenarians. Those undergoing VT ablation were more likely to have complications and a higher mortality rate. Baseline comorbidities can be used to risk-stratify patients when deciding on the best treatment strategy.

## Introduction

Over the past several decades, catheter ablation has emerged as a therapeutic option for patients with commonly encountered arrhythmias. With an ever-increasing population of elderly patients, the burden of arrhythmias, particularly atrial fibrillation (AF), continues to grow.^1^ However, as with any invasive procedure, catheter ablation procedures present their own inherent risks.^2^ As such, multiple studies have investigated the safety and efficacy of ablation in the elderly as compared to a younger population. Limited data on the safety, complications, and mortality across ablations for different arrhythmias in the elderly exist, and the drivers of complications in this delicate population are poorly elucidated. In this study, we used the National Inpatient Sample (NIS) database to examine the safety and outcomes of ablation therapy in elderly patients undergoing treatment for AF, atrial flutter (AFL), supraventricular tachycardia (SVT), and ventricular tachycardia (VT) compared to a younger subgroup.

## Methods

The data used in this study were obtained from the NIS database for the years 2016–2019. The NIS is a publicly available database of hospital discharges in the United States (US), offering data from approximately 8 million hospital stays that were selected using a complex probability sampling design and the weighting scheme recommended by the Agency for Healthcare Research and Quality, which is intended to represent all discharges from non-federal hospitals. It is the largest all-payer inpatient dataset in the US and includes a sample of US community hospitals that approximates 20% of all US community hospitals. Each entry includes one primary diagnosis and up to 39 secondary diagnoses. It also contains information on demographic details, including age, sex, race, insurance status, primary and secondary procedures, hospitalization outcome, total cost, and length of stay (LOS). The NIS database contains clinical and resource use information, with safeguards to protect the privacy of patients, physicians, and hospitals. The NIS database results have been shown to correlate well with other hospitalization discharge databases in the US.

### Study population

After weighing the data, we identified patients aged ≥80 years who were diagnosed with AF, AFL, SVT, and VT as a primary diagnosis using the International Classification of Diseases, Tenth Revision, Clinical Modification (ICD-10-CM). We also identified patients who underwent radiofrequency catheter ablation during the index hospitalization. Afterward, we excluded procedures that may have led to complications that can be attributed to catheter ablation, such as pacemaker or implantable cardioverter-defibrillator insertion, and open surgical ablation. The institutional review board approval was exempted from full review for the current study because all data collection was derived from a de-identified administrative database. The study of catheter ablation for arrhythmias using the NIS database has been validated in previous studies.

### Study variables and outcomes

We extracted all patients aged ≥21 years who were admitted for AF, AFL, SVT, and VT and underwent radiofrequency catheter ablation between the years 2016 and 2019. We extracted each patient’s baseline characteristics from the NIS dataset, including age, sex, ethnicity, hospital size, and hospital status (teaching vs. non-teaching). We used the ICD-10-CM to identify associated comorbidities that might influence the primary outcome of the study, such as congestive heart failure (CHF), coronary artery disease (CAD), chronic obstructive pulmonary disease (COPD), smoking, hyperlipidemia (HLD), peripheral vascular disease (PVD), hypertension, chronic kidney disease (CKD), and multiple other comorbidities included in **Table 1**. We defined the severity of comorbid conditions using Charlson’s comorbidity index (CCI).^3^ We also extracted the common complications of catheter ablation procedures, which were grouped to study the patients’ characteristics associated with developing complications during hospitalization. Patients were divided into two groups: a younger population aged <80 years and an elderly population aged ≥80 years. The primary outcome was all-cause mortality. The secondary outcomes were to assess the LOS and in-hospital complications listed in **Table 2**. Other outcomes studied were independent factors associated with mortality and complications.

**Table 1:** Baseline Characteristics of Study Participants Undergoing Ablation Dichotomized by Age

	Age < 80 Years	Age ≥ 80 Years	*P* Value
Number of patients	150,045	18,595	–
Age (mean ± SD), years	62.7 ± 0.11	84.2 ± 0.05	<.001
Female sex	36.9	55.2	<.001
Race/ethnicity			
White	77	85.3	<.001
Black	11	5.7	<.001
Hispanic	6.8	5.2	<.001
Teaching hospital	86.1	82.8	.002
CCI ≥ 3 points	31	43.61	<.001
Comorbidities			
Chronic kidney disease	17	32.3	<.001
Congestive heart failure	39.8	55.5	<.001
Chronic obstructive pulmonary disease	13.6	16.3	<.001
Coronary artery disease	33	44.3	<.001
Obstructive sleep apnea	20.3	9.2	<.001
Diabetes	28.8	26	<.001
Hypertension	39.9	35.6	<.001
Hyperlipidemia	43.1	53.2	<.001
Coronary artery bypass graft	9.4	14.6	<.001
Anemia	7.6	12.5	<.001
Peripheral vascular disease	3.3	5.7	<.001
Obesity	25.3	9.1	<.001
Smoking	27.9	30.4	.0022

*Abbreviation:* CCI, Charlson’s comorbidity index.

**Table 2:** Mortality, Length of Stay, and Complications of Study Participants Undergoing Ablation Dichotomized by Age

	Age < 80 Years	Age ≥ 80 Years	*P* Value
Mortality (%)	0.7	1.2	.0004
LOS (mean), days	4.2	5.1	<.001
Rehabilitation transfer (%)	4.7	19	<.001
Myocardial injury (%)	2.6	2.9	.29
Pericardial effusion (%)	2.9	2.6	.09
Tamponade (%)	1.1	0.8	.11
Acute pericarditis (%)	0.4	0.06	<.001
Atrioventricular fistula (%)	0.07	0.06	.9
Respiratory failure (%)	0.5	0.3	.045
Post-procedure stroke (%)	0.11	0.04	.15
Post-procedure hemorrhage (%)	0.3	0.2	.6

*Abbreviation:* LOS, length of stay.

### Statistical analysis

The data were expressed as weighted mean ± standard deviation values, and frequencies were denoted in percentages. Independent *t* tests were used to compare continuous variables measurements, while Fisher’s exact tests were used for categorical variables. Weighted values of patient-level observations were generated to produce a nationally representative estimate of the entire US population of hospitalized patients. Univariate and multivariate logistic regression analyses were used to study the association between catheter ablation and the primary and secondary outcomes. The regression model was adjusted for demographics (age, race, and sex), including CCI, other relevant medical comorbidities, patients’ insurance, and hospital characteristics. Linear regression models were used to assess the LOS. Log transformation of LOS was performed to adjust for positively skewed data. We investigated the predictors of mortality and in-hospital complications in patients who underwent catheter ablation using multivariable logistic regression. *P* < .05 was considered statistically significant. We used StataCorp 2017 (StataCorp LLC, College Station, TX, USA) for analysis.

## Results

Over the 4-year period from 2016 to 2019, a total of 168,640 patients were analyzed **(Table 1)**. A total of 150,045 patients aged <80 years with an average age of 62.7 years and 18,595 patients aged ≥80 years with an average age of 84.2 years were included, respectively. Most elderly patients were women (55.2%) and White (85.3%). Comorbidities were significantly more common among the elderly patients, who had higher incidence rates of a CCI score of ≥3 points, CKD, CHF, COPD, CAD, obstructive sleep apnea, type 2 diabetes, hypertension, HLD, coronary artery bypass grafting (CABG), anemia, PVD, and obesity **(Table 1)**. Overall mortality was significantly higher among elderly patients (0.7% vs. 1.2%; *P* = .0004), and their mean LOS was also significantly higher at 5.1 versus 4.2 days (*P* < .001) **(Table 2)**. Rehabilitation transfer was also significantly more common among elderly patients at 19% versus 4.7% (*P* < .001) **(Table 2)**. Rates of procedure-related complications, including myocardial injury, pericardial effusion, tamponade, atrioventricular (AV) fistula, stroke, and hemorrhage, were not significantly different between both the groups. Acute pericarditis was significantly more common in the younger group compared to the elderly one (0.4% vs. 0.06%; *P* < .001) **(Table 2)**.

Among the elderly patients undergoing ablation, the most common procedure performed was ablation for AF (46%, n = 8630), followed by AFL ablation (23%, n = 4305), VT ablation (18%, n = 3400), and SVT ablation (12%, n = 2260) **(Table 3)**. VT ablation patients were more likely to have CAD (74%), CHF (74%), CKD (46%), diabetes (33%), and HLD (63%). CCI scores were higher in the VT ablation group, with 71% of VT ablation patients having a CCI score of ≥3 points compared to 42% in the AFL ablation group, 41% in the AF ablation group, and 37% in the SVT ablation group **(Table 3)**.

**Table 3:** Baseline Demographics of Elderly Patients ≥80 Years Who Underwent Catheter Ablation

	Atrial Fibrillation	Atrial Flutter	Ventricular Tachycardia	Supraventricular Tachycardia
Number of ablations	8630	4305	2260	3400
Age (mean ± SD), years	83.8 ± 0.11	83.9 ± 0.11	83.1 ± 0.15	84.2 ± 0.14
Male sex	39	55.5	86.5	44.1
Race/ethnicity				
White	87.8	80.9	86.4	75.1
Black	3.5	10.1	3.9	11.3
Hispanic	5.4	5.1	4.7	7.8
Teaching hospital	81	82.8	88.5	82.7
Comorbidities (%)				
Coronary artery disease	45.4	43.1	74.1	39.7
Obesity	10.3	9.9	10.8	7.2
Congestive heart failure	58.2	46.5	74.3	29
Chronic kidney disease	29.6	31.7	46	27.8
Diabetes	25.1	28.8	32.7	30.4
Peripheral vascular disease	5.3	4.8	7.5	4.3
Chronic obstructive pulmonary disease	16.3	15.9	19.9	16.3
Hypertension	35.2	37.3	25.4	49.6
Coronary artery bypass graft	13.6	17.9	35	11.6
Anemia	11.9	11.5	14.2	11.9
Smoking	31.1	30.7	33.9	30.6
Alcohol	5.8	5.3	8	5.0
Hyperlipidemia	53.1	50.2	63.3	56.2
Obstructive sleep apnea	11.4	8.9	11.3	6.9
CCI score, points				
0	15.8	17.0	5.1	21.5
1–2	42.9	41.6	23.8	41.1
≥3	41.3	41.5	71.0	37.4

*Abbreviation:* CCI, Charlson’s comorbidity index.

In the elderly subgroup, the average LOS was 6.4 ± 0.21 days among those undergoing VT ablation, while it was 4.8 ± 0.15 days in the AFL group, 4.54 ± 0.11 days in the AF group, and 4.1 ± 0.14 days in the SVT group **(Table 4)**. The most common post-procedure complication in the AF ablation group was AV block (8.8%). The most significant post-procedure complications in the VT ablation group were myocardial injury (3.8%), pericardial effusion (3.5%), and post-procedure AV block (3.5%). Post-procedure myocardial injury occurred in 7.2% of patients in the SVT ablation group—the highest rate among the respective ablation strategies. Acute pericarditis, AV fistula, pneumothorax, respiratory failure, and stroke all occurred <1% of the time across all groups. No patients included in the analysis experienced post-procedure CHF, cardiogenic shock, cardiac arrest, bleeding requiring blood transfusion, or pneumonia **(Table 4)**.

**Table 4:** Complications Following Catheter Ablation in Elderly Patients ≥80 Years Old Who Underwent Catheter Ablation

	Atrial Fibrillation	Atrial Flutter	Ventricular Tachycardia	Supraventricular Tachycardia
Complications (%)				
Myocardial injury	1.6	2.9	3.8	7.2
Pericardial effusion	3.0	2.2	3.5	1.2
Tamponade	0.9	0.7	1.6	0.44
Acute pericarditis	0.058	0.12	*	*
AV fistula	0.05	*	0.44	*
Pneumothorax	0.05	*	0.22	*
Bleeding	0.35	0.12	*	*
Respiratory failure	0.6	0.12	0.22	*
Stroke	*	0.12	*	*
Post-procedure atrioventricular block	8.8	2.3	3.5	1.8
Mortality (%)	0.99	1.3	4.2	0.3
LOS (mean), days	4.54 ± 0.11	4.8 ± 0.15	6.4 ± 0.21	4.1 ± 0.14
Rehabilitation transfer (%)	17.2	15.3	15.3	16.3

*Abbreviation:* LOS, length of stay. *Number of events was <10.

When comparing those who experienced at least one post-procedure complication versus those without any post-procedure complications among all ablation subsets in the elderly group of patients, those experiencing complications were more likely to have CHF (65% vs. 53%; *P* ≤ .001), CKD (40% vs. 35%; *P* = .03), PVD (8% vs. 6%; *P* = .05), and a CCI score of ≥3 points (57.5% vs. 48%; *P* < .001). Fewer post-procedure complications were noted in patients with a history of CABG (13.3% vs. 17%; *P* = .02) **(Table 5)**. Patients experiencing at least one post-procedure complication were more likely to have a longer LOS (7.6 ± 0.25 vs. 5.4 ± 0.07 days; *P* ≤ .001) and more likely to be transferred to acute rehabilitation (26% vs. 20%; *P* = .003) **(Table 5)**.

**Table 5:** Baseline Characteristics and Comorbidities of Elderly Patients ≥80 Years Undergoing Ablation Who Developed Complications Versus No Complications

	≥1 Complication	No Complications	*P* Value
Female sex	7.1	42.0	.33
Race/ethnicity			
White	82.7	84	
Black	6.7	6.5	.2
Hispanic	7.6	5.5	
Comorbidities			
Coronary artery disease	48.9	47.4	.51
Obesity	8.1	9.6	.26
Congestive heart failure	65	53	<.001
Chronic kidney disease	39.5	34.8	.03
Diabetes	30	28.6	.51
Peripheral vascular disease	7.6	5.5	.057
Chronic obstructive pulmonary disease	15.3	16.8	.33
Hypertension	25.1	34.4	<.001
Coronary artery bypass graft	13.3	17	.02
Anemia	15.7	13.5	.14
Smoking	29.3	31.2	.36
Alcohol	8.1	5.9	.05
Hyperlipidemia	52.8	54.1	.56
Obstructive sleep apnea	10.4	9.9	.78
CCI score, points			
0	7.4	14.4	<.001
1–2	34.9	37.2	<.001
≥3	57.5	48	<.001
Transfer to rehabilitation (%)	25.6	20.1	.003
Mortality (%)	0.55	1.2	<.001
LOS (days) (mean ± SD)	7.6 ± 0.25	5.4 ± 0.07	<.001

*Abbreviations:* CCI, Charlson’s comorbidity index; LOS, length of stay.

Pooled mortality was 1.7% among the entire elderly cohort, with the VT ablation group exhibiting the highest mortality (4.2%) **(Table 4)**. After multivariable logistic regression analysis, only a CCI score of ≥3 points (odds ratio [OR], 14.7; 95% confidence interval [CI], 1.88–114.9; *P* = .01) and tamponade (OR, 4.9; 95% CI, 1.65–14.8; *P* = .004) were independent predictors of mortality **(Table 6)**. Factors independently associated with a decreased risk of mortality across ablation groups were female sex (OR, 0.57; 95% CI, 0.34–0.93; *P* = .03) and obesity (OR, 0.09; 95% CI, 0.01–0.7; *P* = .02) **(Table 6)**.

**Table 6:** Independent Predictors of Mortality Following Catheter Ablation in Patients ≥80 Years Old

	Odds Ratio	Confidence Interval	*P* Value
Female sex	0.57	0.34–0.93	.03
CCI score, points			
1	5.8	0.68–49	.11
2	8.4	1.01–68.5	.048
≥	14.7	1.88–114.9	.01
Obesity	0.09	0.01–0.7	.021
Congestive heart failure	1.7	1.1–3.1	.04
Tamponade	4.9	1.65–14.8	.004
Complications	2.2	1.3–3.9	.002

*Abbreviation:* CCI, Charlson’s comorbidity index.

Only CCI scores of ≥2 points were found to be independent predictors for complications following any ablation (AF, AFL, SVT, and VT) in the elderly, with a CCI score of ≥3 points having the greatest significance (OR, 2.14; 95% CI, 1.4–3.3; *P* = .001) **(Table 7)**.

**Table 7:** Independent Predictors of Complications Following Catheter Ablation in Patients ≥80 Years

	Odds Ratio	Confidence Interval	*P* Value
CCI score, points			
1	1.5	1.0–2.2	.048
2	1.7	1.1–2.5	.009
≥3	2.14	1.4–3.3	.001
Congestive heart failure	1.2	1.1–1.8	.03
Chronic kidney disease	0.82	0.63–1.05	.08
Chronic obstructive pulmonary disease	0.76	0.6–0.99	.03

*Abbreviation:* CCI, Charlson’s comorbidity index.

## Discussion

In this study using the NIS database, we were able to identify a total of 168,640 patients undergoing ablation therapy for either AF, AFL, VT, or SVT. The elderly subgroup had significantly higher incidences of comorbidities, and their mortality rate was also significantly greater at 1.2% versus 0.7% (*P* = .0004). We also found that the length of hospitalization and transfer to rehabilitation on discharge were significantly higher in the elderly population. Acute pericarditis was the only post-procedure complication noted to be significant and was higher in the younger population compared to the elderly one. Elderly patients undergoing VT ablation were more likely to have CAD, CHF, CKD, diabetes, and the highest CCI score compared to patients undergoing ablation for other indications. Overall mortality rate in the elderly population was driven by those undergoing VT ablation, which was highest at 4.2%, and the LOS was also greatest in this group at 6.4 days. Independent predictors of mortality in elderly patients undergoing catheter ablation were CCI scores of >2 points (OR, 8.4 ± 1.01–68.5; *P* = .048), CCI scores of ≥3 points (OR, 14.7 ± 1.88–114.9; *P* = .01), and procedure-related tamponade (OR, 4.9 ± 1.65–14.8; *P* = .004).

Similar to findings in our study, older age has been shown by others to increase the risk of mortality in patients undergoing ablation therapy. In a study by Nademanee et al., the safety and efficacy of catheter ablation in patients older than 75 years of age were evaluated. A total of 261 patients underwent ablation, and the average age was 79 years. Achieving normal sinus rhythm was an independent factor associated with better outcomes, particularly survival. However, older age and low ejection fraction were independent predictors of mortality. Female sex, hypertension, and CHF were not independently associated with increased mortality.^1^ In another retrospective study, the outcomes and safety of catheter ablation of VT in octogenarians were evaluated. Here, patients over the age of 80 years were compared with younger patients, and the analysis included a total of 54 consecutive patients with an average age of 82.8 years compared to a control group of 104 patients with an average age of 66.7 years. Ultimately, investigators Frontera et al. found that a higher rate of major complications occurred in octogenarians (18% vs. 2%), driven by cardiac tamponade, AV block, and pseudoaneurysm.^4^ In a separate analysis of patients separated by the age of 70 years, major complications were more frequent in the elderly (5.3% vs. 3.2%; *P* = .03); however, this was driven by vascular complications. There were similar rates of cerebrovascular and non-vascular complications between both age groups,^5^ similar to findings in our study. A meta-analysis evaluated the long-term efficacy and safety of ablation in elderly patients with AF and involved a total of 20 observational studies with 8009 patients. As compared to the non-elderly, elderly patients had significantly more major complications and overall complications with greater rates of cerebrovascular events.^6^ In our analysis, patients >80 years old were found to have significantly higher incidences of comorbidities, as would be expected. Additionally, overall mortality was significantly longer among elderly patients (0.7% vs. 1.2%; *P* = .0004), and the mean LOS was also significantly longer among the elderly patients at 5.1 versus 4.2 days (*P* < .001) **(Table 2)**. However, rates of complications, which included myocardial injury, pericardial effusion, tamponade, AV fistula, and post-procedure stroke/hemorrhage, were not significantly different between the groups **(Table 2)**. This is similar to the results of a meta-analysis on the efficacy and safety of ablation for symptomatic AF in elderly patients (>75 years old), which showed a similar incidence of complications after AF ablation in patients <60 years.^7^ In our study, the only significant difference in post-procedure complications was acute pericarditis, which was higher in our younger subgroup (0.4% vs. 0.06%). Given the nature of the NIS database, we were unable to further evaluate why this complication was noted in the younger subgroup, but we postulate that clinicians are more likely to be aggressive with their ablation parameters and strategies in a younger population, which may inadvertently lead to higher incidence rates of pericarditis.

The mortality rate in our elderly population was primarily driven by patients undergoing VT ablation, who, in our subgroup analysis, were found to have the highest mortality rate at 4.2% **(Table 4)**. Patients undergoing VT ablation were also noted to have a higher incidence of comorbidities, including CAD, CHF, type 2 diabetes, CABG history, and a CCI score of ≥3 points **(Table 3)**. After multivariable logistic regression analysis, only a CCI score of ≥3 points (OR, 14.7; 95% CI, 1.88–114.9; *P* = .01) and tamponade (OR, 4.9; 95% CI, 1.65–14.8; *P* = .004) were independent predictors of mortality in the elderly **(Table 6)**. A study on 2049 patients undergoing VT ablation at 12 centers showed that patients ≥70 years had a higher in-hospital mortality (4.4% vs. 2.3%; *P* = .01).^8^ This is similar in outcome to the mortality rate in the VT ablation subgroup in our study. In a study by Willy et al. on ablation outcomes in the very elderly, patients undergoing ablation for AFL, AV nodal reentrant tachycardia (AVNRT), or VT were enrolled, and procedural outcomes were compared with matched groups aged 60–80 years and aged 40–60 years. The analysis included 1191 patients, and acute success was high in all groups. The rate of periprocedural complications was similar in all groups for AFL and AVNRT, but the rate of complications was higher in patients undergoing VT ablation. Most common complications include infections and groin hematoma.^9^ These findings are similar to the results of our study, in which a higher rate of complications in the VT subgroup was noted, likely secondary to a sicker subgroup with more cardiovascular risk factors. Additionally, we found that the average LOS was highest in the VT group (6.4 ± 0.21 days) **(Table 4)**. This is similar to the study by Frontera et al., who evaluated outcomes of catheter ablation of VT in octogenarians compared to younger patients and found the elderly group to have more risk factors and longer lengths of hospitalization (9.1 vs. 6.2 days).^4^

The elderly subgroup of patients in our study who experienced post-procedure complications were more likely to have CHF (65% vs. 53%; *P* ≤ .001), CKD (40% vs. 35%; *P* = .03), PVD (8% vs. 6%; *P* = .05), and a CCI score of ≥3 points (*P* ≤ .001) **(Table 5)**. Fewer complications were noted in patients with a history of CABG. This can be explained by fibrotic changes to the pericardial sac in patients with a history of CABG, which may lead to a lower incidence of perforation and resultant tamponade. Only CCI scores of ≥2 points were found to be independent predictors for complications following any ablation (AF, AFL, SVT, and VT) in the elderly, with a CCI score of ≥3 points carrying the greatest significance (OR, 2.14; 95% CI, 1.4–3.3; *P* = .001) **(Table 7)**. In a prospective study on the incidence and predictors of major complications from catheter ablation procedures, Bohnen et al. found that ablation for AF or VT and serum creatinine level of >1.5 mg/dL was associated with an increased risk of major complications.^10^ In another study on the predictors of complications of catheter ablation for AF, 1295 patients were analyzed, and female sex and undergoing ablation in July or August were found to be independent predictors of any complication.^11^ Elsewhere, a higher CHA_2_DS_2_-VASc score and early institutional experience were independent predictors of adverse events in a separate study on elderly patients undergoing AF ablation.^12^ These findings are similar to our study, where elderly patients with post-procedure complications were more likely to have underlying vascular disease, CHF, and CKD. As would be expected, a longer LOS was seen in patients who experienced at least one post-procedure complication (7.6 ± 0.25 vs. 5.4 ± 0.07 days; *P* ≤ .001). Additionally, these patients were more likely to be transferred to acute rehabilitation (26% vs. 20%; *P* = .003) **(Table 5)**.

The most common post-procedure complication in the AF ablation group was AV block (8.8%). Post-procedure myocardial injury occurred in 7.2% of patients in the SVT ablation group, the highest of the respective ablation strategies. Based on the nature of NIS data, we were unable to determine the degree of AV block and whether it was transient in nature following ablation or required pacemaker insertion. Conduction block after radiofrequency energy application may be transient, and this complication may not be directly related to the procedure itself.^13^ Similarly, we were unable to accurately define myocardial injury in the database and whether it was secondary to a demand-type ischemia in patients undergoing SVT ablation or acute coronary syndrome requiring further intervention. Acute pericarditis, AV fistula, pneumothorax, respiratory failure, and stroke all occurred <1% of the time across all ablation groups. No patients included in the analysis experienced post-procedure CHF, cardiogenic shock, cardiac arrest, bleeding requiring blood transfusion, or pneumonia **(Table 2)**. Factors independently associated with decreased risk of mortality across ablation groups were female sex (OR, 0.57; 95% CI, 0.34–0.93; *P* = .03) and obesity (OR, 0.09; 95% CI, 0.01–0.7; *P* = .02) **(Table 6)**. The correlation between female sex and mortality outcomes in our analysis is unclear. Some studies have reported increased trends toward mortality associated with female sex,^14^ and others have cited increased rates of complications.^15,16^ However, others have noted no differences in complication rates related to sex, similar to our analysis.^1,17^ A recent study found no significant impact of obesity on complication rates of catheter ablation. In our study, decreased mortality was noticed in obese patients, likely due to reduced rates of complications (particularly perforation/tamponade) secondary to the prominence of pericardial adipose tissue.

### Limitations

Several limitations were identified in our study. Our analysis is based on retrospective data obtained from administrative databases. As such, we are unable to extrapolate additional data on details pertaining to complications or outcomes. Additionally, the NIS database does not include long-term outcomes and is only limited to in-hospital outcomes. We are also only able to make observations based on correlation, which does not explain causation.

## Conclusion

With an ever-increasing elderly population, the burden of arrhythmias will continue to rise. Ablation therapy has become an attractive option for a variety of different arrhythmias, but this invasive procedure comes with its own inherent risks. In this study, we found a higher mortality rate in elderly patients compared to a younger population, along with an increased rate of transfer to rehabilitation and mean length of hospitalization. The mortality rate in the elderly subgroup was primarily driven by a higher incidence of mortality in patients undergoing VT ablation. No procedural-related complications were noted in either group. Given these data, candidacy for ablation therapy should be based on individual risk profile, and in-depth communication with patients prior to intervention should be had.
